# Exploratory Shotgun Metagenomic Analysis of the Fecal Microbiome and Antimicrobial-Resistance Potential in Simmental Calves with Different Diarrheal Statuses

**DOI:** 10.3390/microorganisms14102370

**Published:** 2026-10-16

**Authors:** Mengyuan Xie, Hewei Shang, Fengwu Wang, Jiangliu Yang, Nali Geer, Qian Zhang, Xiaojing Xu, Dalai Baolige

**Affiliations:** 1 College of Veterinary Medicine, Inner Mongolia Agricultural University, Hohhot 010011, China; 18330018791@emails.imau.edu.cn (M.X.); shw@emails.imau.edu.cn (H.S.); 2 Key Laboratory of Clinical Diagnosis and Treatment Technology in Animal Disease, Ministry of Agriculture and Rural Affairs, Hohhot 010010, China; 3 Veterinary Research Institution, Inner Mongolia Academy of Agricultural and Animal Husbandry Sciences, Hohhot 010031, China

**Keywords:** calf diarrhea, fecal microbiome, *Enterobacteriaceae*, microbial functional potential

## Abstract

Calf diarrhea is frequently accompanied by disruption of the gastrointestinal microbiota, but the relationships between clinical status and microbial community composition, gene-content functional potential, and antimicrobial-resistance-associated features remain incompletely characterized. This exploratory study aimed to characterize these features in calves with different diarrheal statuses using shotgun metagenomics. Six approximately 2-month-old Simmental calves from Chahar Right Middle Banner, Inner Mongolia, were included, comprising two healthy calves, two calves with mild diarrhea, and two calves with severe diarrhea. Read-based taxonomic profiling was complemented by gene-abundance-weighted taxonomic annotation, and microbial functional potential was characterized using eggNOG/COG, KEGG, and CAZy annotations, together with conservatively screened CARD-associated sequence-homology candidates. The two severe-diarrhea samples exhibited lower bacterial richness and Shannon diversity, although PCoA did not show clear separation according to clinical severity. These two samples showed high representation of Proteobacteria and *Enterobacteriaceae*-related taxa, particularly Escherichia-related assignments, together with lower representation of *Ruminococcaceae*, *Faecalibacterium*, and *Prevotella*. Gene-content profiles showed higher representation of selected functions related to transport, environmental sensing, motility, secretion, and bacterial cell-envelope processes, whereas several glycan- and complex-carbohydrate-associated functions showed lower representation. Selected CARD-associated candidates were also more abundant in the severe-diarrhea samples. Within this small exploratory cohort, the two severe-diarrhea samples therefore showed concordant taxonomic and gene-content differences; however, these observations should not be interpreted as a general severity-specific microbiome signature and require validation in larger longitudinal cohorts.

## 1. Introduction

Calf diarrhea remains a major cause of morbidity, mortality, impaired growth, and economic loss during the preweaning period. Its etiology is multifactorial and involves interactions among enteric pathogens, host immunity, nutrition, management, and environmental conditions [1,2]. In addition to recognized infectious agents, increasing attention has been directed toward the gastrointestinal microbiota because microbial colonization during early life contributes to nutrient utilization, development of mucosal immunity, colonization resistance, and maturation of the gastrointestinal tract [3,4]. The calf gut microbiota develops rapidly after birth and undergoes pronounced age-dependent changes before and around weaning, with substantial variation among individual animals [5]. These characteristics make the early preweaning period a particularly dynamic stage in which disruption of microbial community development may be associated with gastrointestinal disease.

Accumulating evidence indicates that calf diarrhea is accompanied by marked alterations in fecal microbial diversity and community composition. Reduced bacterial diversity has been associated with diarrhea in preweaned calves, and several studies have reported changes involving *Faecalibacterium*, *Ruminococcaceae*, *Prevotella*, *Enterobacteriaceae*, and *Escherichia/Shigella*-related taxa [6,7,8,9]. Gomez et al. further described diarrhea-associated shifts from obligate toward facultative anaerobic populations, whereas longitudinal studies have shown that microbial diversity and community structure may change around the onset of diarrhea and partially recover after clinical resolution [9,10]. Considerable heterogeneity nevertheless exists among animals and farms, indicating that diarrhea is unlikely to correspond to a single universally conserved taxonomic profile. Age, management, diet, environmental exposure, and the underlying cause of diarrhea may all contribute to the observed variation [6,7,8,9,10].

Most early studies of the calf fecal microbiota relied primarily on 16S rRNA gene sequencing and therefore focused on bacterial community composition. More recent shotgun metagenomic and multi-omic investigations have shown that diarrhea-associated dysbiosis can also involve substantial changes in microbial gene content and metabolic potential. Whon et al. linked calf diarrhea with prolonged expansion of gut *Enterobacteriaceae* and accompanying functional dysbiosis [11], while integrated metagenomic and metabolomic analysis demonstrated that taxonomic changes in diarrheic calves were accompanied by alterations in microbial metabolic functions and metabolites [12]. A recent multi-farm study from Inner Mongolia further highlighted the complexity of diarrhea-associated microbiome restructuring across different clinical severities using both 16S and metagenomic approaches [13]. In parallel, metagenomic studies have shown that young calves harbor a dynamic fecal resistome closely associated with microbiota succession and early-life diet [14]. Antimicrobial exposure is another important factor shaping the early-life calf microbiome. Young calves can harbor diverse antimicrobial-resistance genes, and both antimicrobial treatment and changes in microbial community composition may influence fecal resistome profiles. The bovine intestinal microbiome may therefore serve as a reservoir of resistance-associated genetic determinants with relevance to animal health and antimicrobial stewardship. Shotgun metagenomics therefore provides an opportunity to examine the calf fecal microbiome beyond taxonomic composition by integrating community structure with gene-level functional potential and antimicrobial-resistance-associated features [15]. However, despite increasing metagenomic evidence of diarrhea-associated dysbiosis in calves, the relationships among taxonomic composition, gene-content functional potential, carbohydrate-active enzyme profiles, and antimicrobial-resistance-associated features within the same fecal metagenomes remain incompletely characterized, particularly at the individual-sample level. Integrating these complementary profiles may provide a more comprehensive description of the microbial ecological changes accompanying calf diarrhea and help identify patterns that warrant validation in larger cohorts.

In this study, shotgun metagenomic sequencing was used to characterize fecal microbial communities from healthy, mildly diarrheic, and severely diarrheic Simmental calves. Read-based taxonomic profiling was complemented by gene-abundance-weighted taxonomic annotation, while microbial functional potential was evaluated using eggNOG/COG and KEGG annotations, carbohydrate-active enzyme profiles were characterized using CAZy, and antimicrobial-resistance-associated potential was explored using conservatively screened CARD-associated sequence-homology candidates. This exploratory study aimed to determine whether the six sampled calves with different diarrheal statuses showed corresponding differences in fecal microbial community composition and gene-content functional potential, with particular attention to the two severe-diarrhea samples.

## 2. Materials and Methods

### 2.1. Main Reagents and Instruments

The following reagents and instruments were used: E.Z.N.A. stool DNA Kit (D4015-00S) (Omega Bio-Tek, Norcross, GA, USA); TruSeq™ DNA Sample Prep Kit (Illumina, San Diego, CA, USA); phosphate buffered saline (HyClone, Logan, UT, USA); Covaris M220 Focused-ultrasonicator (Covaris, Woburn, MA, USA).

### 2.2. Sample Collection and Treatment History

Fecal samples were collected from the dung of Simmental calves grazing in Chahar Right Middle Banner, Ulanqab, Inner Mongolia. Six calves were included in the study, comprising two healthy calves (H1 and H2), two calves with mild diarrhea (M1 and M2), and two calves with severe diarrhea (S1 and S2). All calves were approximately 2 months old and weighed 30 ± 2 kg according to the original sampling records. Calves within a similar age and body-weight range were selected to reduce age-related variation in the developing gut microbiota and improve comparability among the sampled animals. Clinical grouping was based on fecal consistency recorded at the time of sampling. Healthy calves had normally formed feces without visible signs of diarrhea; calves with mild diarrhea had loose or mucus-containing feces; and calves with severe diarrhea had watery feces. Farm records indicated that the four diarrheic calves (M1, M2, S1, and S2) had received treatment for diarrhea before sampling, whereas the two healthy calves (H1 and H2) had not received such treatment. Antimicrobial classes used on the farm for calf diarrhea included β-lactams, cephalosporins, aminoglycosides, macrolides, tetracyclines, fluoroquinolones, amphenicols, and lincosamides, together with supportive therapies (Table A1). However, animal-specific information on the antimicrobial administered, dosage, treatment duration, and interval between treatment and sampling was not available from the historical records. Fresh fecal samples were collected rectally using sterile gloves, immediately frozen in liquid nitrogen, and stored at −80 °C until further analysis.

### 2.3. DNA Extraction, Library Preparation, and Sequencing

Genomic DNA was extracted from individual fecal samples using the E.Z.N.A. stool DNA Kit according to the manufacturer’s instructions. DNA integrity was evaluated by 1% agarose gel electrophoresis. Qualified genomic DNA was fragmented to approximately 450 bp using a Covaris M220 Focused-ultrasonicator (Covaris, Woburn, MA, USA), and paired-end sequencing libraries were constructed using the TruSeq DNA Sample Preparation Kit (Illumina, San Diego, CA, USA). Following library amplification and quality assessment, shotgun metagenomic sequencing was performed on an Illumina NovaSeq 6000 platform (Illumina, San Diego, CA, USA) to generate 150 bp paired-end reads.

### 2.4. Sequence Quality Control

Raw paired-end reads were subjected to quality filtering using Trimmomatic v0.39. Adapter sequences and adapter-derived reads were removed, low-quality bases were trimmed, reads containing more than 10% ambiguous nucleotides (N) were discarded, and reads shorter than 75 bp after trimming were excluded. Trimmomatic was run using the parameters ILLUMINACLIP:adapters.fa:2:30:10, SLIDINGWINDOW:4:15, and MINLEN:75. The resulting high-quality reads were retained as clean reads for downstream analyses. Sequencing quality was assessed using the total number of reads and bases, GC content, and the proportions of bases with Phred quality scores ≥ 20 (Q20) and ≥30 (Q30). Read-retention and base-retention rates were calculated as the number of clean reads divided by raw reads and clean bases divided by raw bases, respectively.

A separate host-filtering step was recorded in the original bioinformatic outputs. For each sample, the total input read pairs and the corresponding host and non-host read-pair counts were available, and the host-derived and non-host proportions were calculated relative to the total input read pairs at this stage. The software and bovine reference-genome build used for this host-filtering step were not specified in the original analysis report.

### 2.5. Taxonomic Profiling and Relative-Abundance Calculation

Following quality filtering and host filtering, the retained non-host reads were subjected to taxonomic classification using Kraken2 v2.0.6 with the parameters --threads 16 --quick --report-zero-counts --gzip-compressed --paired. Read-based taxonomic profiles generated by the pipeline were used as the primary source for community-composition analyses. Taxonomic assignments were summarized at the domain, phylum, class, order, family, genus, and species levels. Analyses of bacterial community composition were restricted to reads classified within the bacterial domain. For each sample, the relative abundance of a bacterial taxon was calculated as the number of reads assigned to that taxon divided by the total number of reads classified as Bacteria in the same sample, multiplied by 100. Phylum-, family-, and genus-level read-count matrices were used for taxonomic visualization and focused descriptive comparisons. Individual-sample profiles were retained throughout the analysis rather than averaging the two biological replicates within each clinical category. For stacked-bar visualization, major taxa were displayed individually, and the remaining bacterial fraction was combined as “Others”. Family- and genus-level taxa showing concordant abundance patterns in both severe-diarrhea samples were further examined at the individual-sample level.

### 2.6. Metagenomic Assembly, Gene Prediction, and Gene-Abundance Estimation

Quality-controlled metagenomic reads from each sample were assembled de novo using MEGAHIT v1.1.1-2-g02102e1 with the parameter --min-contig-len 500. Open reading frames (ORFs) were predicted from the assembled contigs using MetaProdigal v2.6.3. Predicted genes from all samples were pooled and clustered using CD-HIT v4.8.1 with the parameters -n 9 -c 0.95 -G 0 -M 0 -d 0 -aS 0.9 -r 0 -T 40, corresponding to 95% sequence identity and 90% coverage of the shorter sequence. The longest sequence within each cluster was retained as the representative sequence to construct the non-redundant gene catalog. The resulting catalog contained 731,877 non-redundant genes. Gene abundance in each sample was estimated using Salmon v1.1.0 with the default settings and used for subsequent taxonomic and functional annotation. Detailed assembly and ORF statistics for individual samples are provided in Table A2 and Table A3. Sample-specific read-mapping rates to the non-redundant gene catalog were not reported in the archived project outputs.

### 2.7. Gene-Centered Taxonomic and Functional Annotation

Predicted protein sequences from the non-redundant gene catalog were annotated against multiple reference databases (Table A3). For gene-centered taxonomic annotation, protein sequences were searched against the NCBI non-redundant protein (NR) database using DIAMOND v0.9.22.123 in BLASTP mode with an E-value threshold of 1 × 10^−5^. Taxonomic information associated with the NR annotations was linked to the corresponding catalog genes, and the abundance estimates of genes assigned to the same taxon were summed to generate gene-abundance-weighted taxonomic profiles at different taxonomic ranks. For the NR-based taxonomic profiles, relative abundance at each taxonomic rank was calculated as the summed normalized abundance of genes assigned to a given taxon divided by the total normalized gene abundance assigned to Bacteria in the same sample, multiplied by 100. The same bacterial denominator was retained across all taxonomic ranks, including genes that could not be resolved to a lower rank.

Functional annotation against eggNOG was performed using eggNOG-mapper v5.0 with the default settings. COG functional categories were subsequently summarized by aggregating the abundance estimates of genes assigned to the corresponding categories. KEGG annotation was performed using KofamScan v1.2.0 with the default settings, and KO assignments were summarized at KEGG Level 1, Level 2, Level 3, and pathway levels. Carbohydrate-active enzyme annotation was performed using HMMER hmmscan v3.3.2 against dbCAN-HMMdb-V8 with the default settings, and gene-abundance estimates were aggregated according to CAZy classes and families.

Gene abundance was expressed as normalized gene-abundance units per million. For each sample, the complete abundance vector across the 731,877 non-redundant catalog genes summed to approximately 1,000,000 units, with only minor rounding differences. The original provider documentation describes gene-length normalization and allocation of mapped reads during abundance estimation, conceptually corresponding to

(1) a_g_ = 10^6^ ×(*c_g_*\*L_g_*)\∑_j_(c_j_\L_j_)

where *c_g_* represents the allocated read count for gene *g* and *L_g_* represents gene length. The original effective-length settings and multi-mapping options were not retained and therefore could not be independently verified. Accordingly, these values are interpreted as normalized metagenomic gene-abundance estimates rather than raw read counts, transcript expression, absolute gene-copy numbers, or copies per gram of feces.

For functional profiles, recovered COG, KEGG, and CAZy values were treated as annotation-based abundance aggregates. Because functional annotations and pathways are not necessarily mutually exclusive, category totals should not be interpreted as components of a single 100% partition. The exact historical apportionment rules for genes with multiple annotations could not be independently reconstructed from the archived outputs.

### 2.8. CARD-Associated Antimicrobial-Resistance Annotation and Conservative Screening

Predicted protein sequences were compared with the Comprehensive Antibiotic Resistance Database (CARD) using BLASTP v2.9.0+ with the parameters -max_target_seqs 5 -evalue 1 × 10^−50^ -outfmt 6 -num_threads 8 (Table A4). The exact CARD database release and access date were not specified in the original project records. The historical CARD analysis was based on BLASTP sequence similarity rather than CARD/RGI model classification; therefore, RGI Perfect, Strict, or Loose model categories were not assigned.

For the revised analysis, amino-acid identity and aligned-length information from the archived CARD output were combined with query-protein lengths obtained from the recovered non-redundant protein catalog. An alignment-length ratio was calculated as

(2) alignment-length ratio (%) = 100 × (reported aligned amino-acid length\query-protein length)

This ratio was used only as a sequence-screening metric and should not be interpreted as CARD reference-protein coverage or coordinate-based query coverage. The archived similar (%) field was not treated as alignment coverage. Candidates were retained when amino-acid identity was ≥99% and the alignment-length ratio was ≥95%. Because sequence identity alone cannot establish mutation-dependent CARD resistance models, a conservative label-based review was applied to exclude 25 query genes associated with mutation-dependent resistance annotations for which the required resistance-associated mutations could not be verified. This label-based exclusion was not equivalent to model-aware CARD/RGI validation and may not identify every mutation-dependent annotation; accordingly, all retained entries were interpreted only as sequence-homology candidates.

To avoid double counting, each retained non-redundant catalog query gene contributed only once to the overall CARD-associated abundance. When multiple retained annotations were present for the same query, records were prioritized by higher amino-acid identity, followed by longer aligned length; the ARO identifier was used only as a deterministic tie-breaker when required. Candidate abundance was obtained from the normalized gene-abundance matrix described above and was therefore expressed in normalized gene-abundance units per million. When multiple distinct retained query genes were assigned to the same CARD reference label, their normalized abundances were summed for that label.

### 2.9. Microbial Diversity, Descriptive Comparative Analysis, and Visualization

Microbial diversity analyses were performed using the read-based bacterial genus abundance matrix. Because sequencing depth differed substantially among samples, alpha-diversity estimates were calculated after repeated rarefaction. Bacterial genus-level read counts were rarefied to 837,313 genus-assigned reads per sample, corresponding to the minimum genus-level sequencing depth among the six samples. Rarefaction was independently repeated 300 times for each sample using multivariate hypergeometric sampling. Observed genus richness and the Shannon diversity index were calculated for each rarefied profile, and the mean value across the 300 iterations was used for visualization and descriptive comparison. For beta-diversity analysis, genus-level bacterial read counts were converted to within-sample relative-abundance profiles. Pairwise Bray–Curtis dissimilarities were calculated among the six samples, and PCoA was performed on the resulting dissimilarity matrix to visualize community-structure variation. The percentages of variation represented by the first two principal coordinates were calculated from the positive eigenvalues of the PCoA solution.

Given the exploratory study design and the limited number of biological replicates (*n* = 2), individual samples rather than group means were treated as the primary analytical units. No formal group-level significance tests were used to define taxonomic or functional differences, and Bray–Curtis dissimilarities were not interpreted as *p* values. For descriptive screening of functional profiles, individual-sample abundance patterns were examined across the six metagenomes. A feature was considered to show a concordant directional pattern in the severe-diarrhea samples when its normalized abundance was higher in both S1 and S2 than in each of H1, H2, M1, and M2 or lower in both S1 and S2 than in each of the four non-severe samples. Features meeting this criterion were further considered for focused visualization according to their annotated biological relevance to the taxonomic and functional patterns examined in this study. This post hoc screening procedure was used only for descriptive visualization and interpretation and was not a prespecified discovery strategy or a statistical significance test.

All recovered broad CAZy classes were retained for class-level comparison, whereas the focused family-level comparison used the same concordant directional criterion described above. Complete six-sample abundance matrices for all recovered COG categories, KEGG Level 1, Level 2, and Level 3 categories/pathways, and CAZy classes and families are provided in Supplementary Tables S1–S7.

eggNOG/COG, KEGG, and CAZy were regarded as complementary annotation frameworks applied to the same metagenomic gene content rather than as independent experimental validation. Differences in gene abundance were interpreted as differences in encoded functional potential or gene-content representation and not as evidence of gene expression, pathway activation, enzyme activity, metabolite production, or metabolic flux. Group means and severe/non-severe abundance ratios reported in Supplementary Tables were used only as descriptive summaries and were not interpreted as inferential fold changes. Concordance between taxonomic and functional restructuring was assessed descriptively by comparing individual-sample patterns across the read-based taxonomic profiles, KEGG pathway profiles, and CAZy family profiles.

## 3. Results

### 3.1. Sequencing and Quality Control

Shotgun metagenomic sequencing generated a total of approximately 86.24 Gb of raw sequence data from the six fecal samples, with individual samples yielding 9.94–19.85 Gb of raw data. After adapter removal and quality filtering, approximately 59.58 Gb of clean sequence data were retained, corresponding to an overall base-retention rate of 69.09%. Individual samples yielded 19.75–114.95 million clean reads and 2.87–16.95 Gb of clean sequence data. The proportions of bases with Phred quality scores of at least Q20 and Q30 ranged from 96.60% to 97.91% and from 88.30% to 93.13%, respectively. Compared with the raw data, for which Q30 values ranged from 85.33% to 92.31%, quality filtering consistently increased the proportion of high-quality bases.

The amount of sequence retained after initial quality filtering varied substantially among samples. Raw-to-clean read-retention rates were 85.89%, 69.87%, 76.09%, 29.78%, 86.85%, and 41.79% for H1, H2, M1, M2, S1, and S2, respectively, with M2 and S2 showing the lowest retention. During this step, reads failing the predefined adapter, sequence-quality, ambiguous-base, or minimum-length criteria were removed. However, the archived quality-control output did not provide sample-specific discard counts for each filtering category; therefore, the lower retention of M2 and S2 could not be attributed quantitatively to any single filtering criterion. Host filtering represented a separate subsequent processing stage. Host-derived read pairs accounted for 15.75%, 54.02%, 53.78%, 73.48%, 30.54%, and 57.97% of the host-filtering input for H1, H2, M1, M2, S1, and S2, respectively, leaving corresponding non-host fractions of 84.25%, 45.98%, 46.22%, 26.52%, 69.46%, and 42.03%. Because host filtering occurred after generation of the clean reads, variation in host-read content does not account for the preceding differences in raw-to-clean retention.

Among the read pairs entering downstream taxonomic classification, bacterial assignments accounted for 45.53%, 41.77%, 36.87%, 34.56%, 80.03%, and 75.09% of H1, H2, M1, M2, S1, and S2, respectively, whereas the corresponding unclassified fractions were 54.05%, 57.96%, 62.74%, 64.79%, 18.65%, and 23.43%. The remaining classified reads represented minor eukaryotic, archaeal, and viral assignments. Detailed stage-specific sequencing statistics are provided in Table A5.

### 3.2. Fecal Microbial Diversity and Community Structure

**Figure 1:**
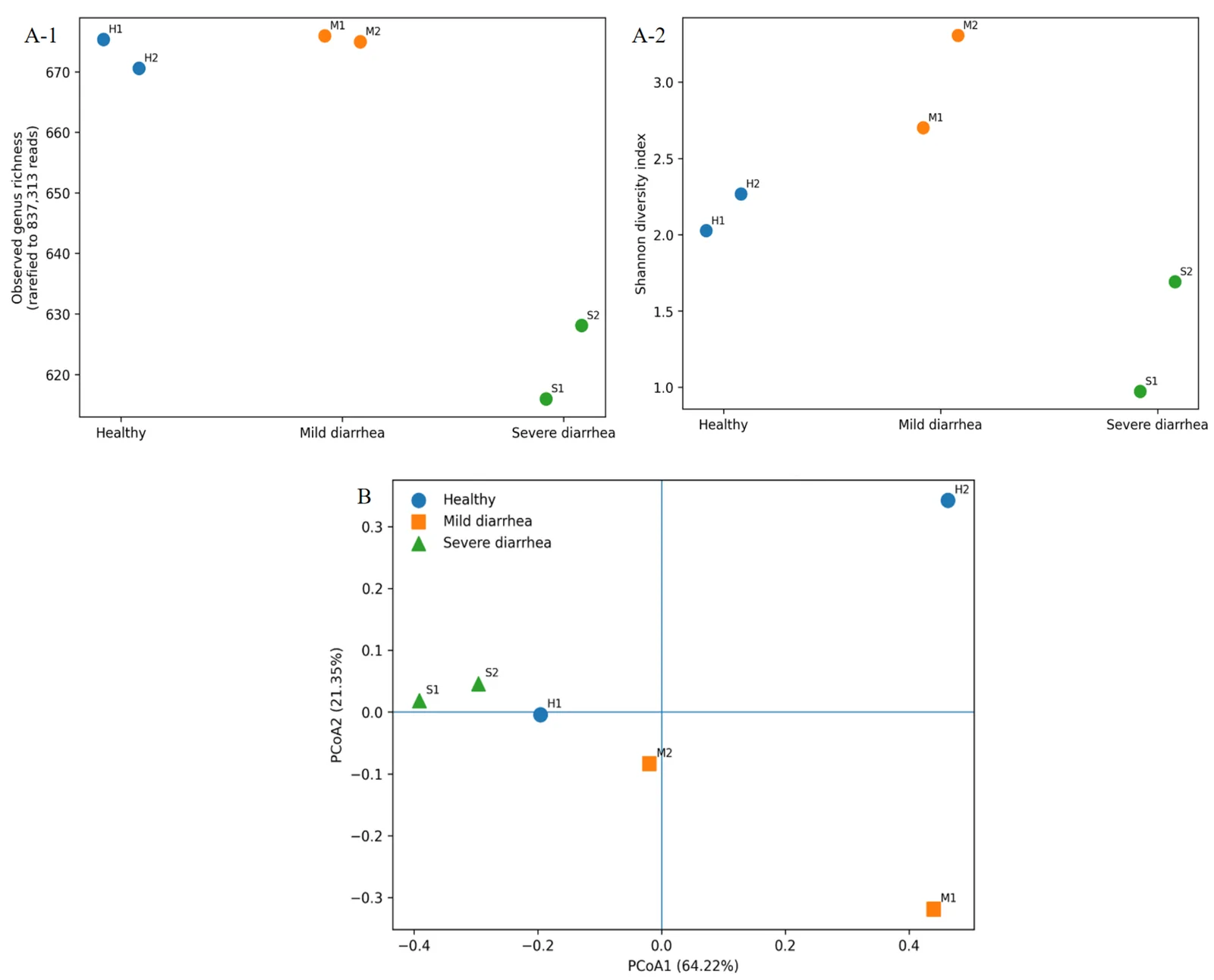
Diversity and community structure of fecal bacterial microbiomes in calves with different diarrheal statuses. (**A-1**,**A-2**) Rarefied observed genus richness and Shannon diversity for individual fecal samples. (**B**) Bray–Curtis PCoA based on genus-level relative-abundance profiles.

**Figure 2:**
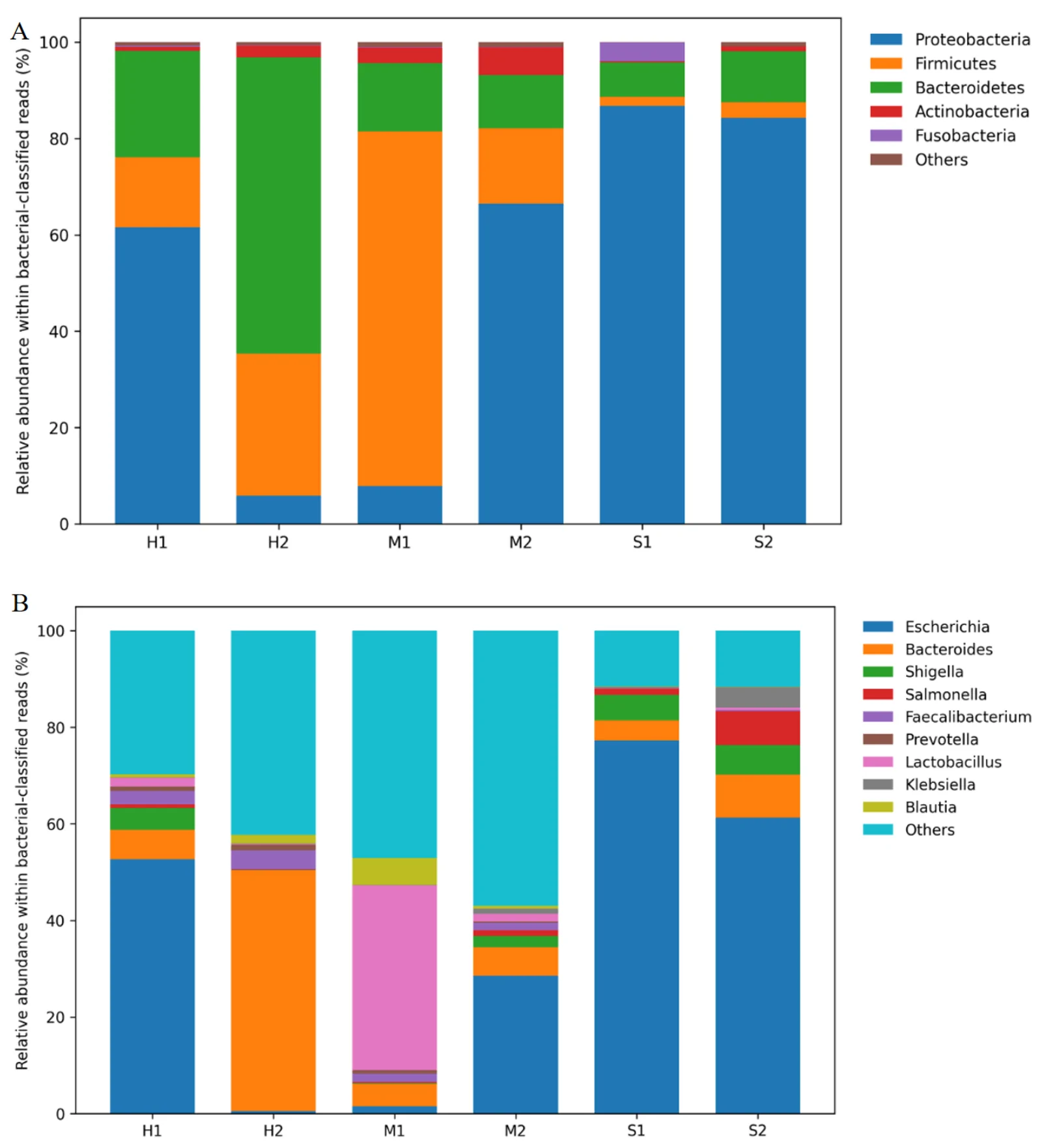
Taxonomic composition of fecal bacterial communities in healthy, mildly diarrheic, and severely diarrheic calves. (**A**) Phylum-level relative abundance of bacterial communities in individual fecal samples. (**B**) Genus-level relative abundance of bacterial communities in individual fecal samples.

To minimize the influence of unequal sequencing depth among samples, bacterial genus-level abundance profiles were rarefied to 837,313 reads per sample before alpha-diversity estimation. Both severe-diarrhea samples exhibited lower rarefied genus richness than the healthy and mildly diarrheic samples, with observed genus richness values of 616.0 and 628.1 for S1 and S2, respectively, compared with 670.6–675.9 among the four non-severe samples (Figure 1(A-1,A-2)). A similar pattern was observed for Shannon diversity, which was 0.974 and 1.692 in S1 and S2, respectively, whereas values in the healthy and mild-diarrhea samples ranged from 2.026 to 3.306. Thus, both severe-diarrhea microbiomes exhibited lower genus-level richness and diversity than the four non-severe microbiomes.

Bray-Curtis dissimilarities calculated from genus-level relative-abundance profiles were subsequently subjected to PCoA. The first and second principal coordinates explained 64.22% and 21.35% of the total variation, respectively, accounting for 85.58% of the variation in community composition (Figure 1B). The ordination did not show clear separation according to clinical severity. Although S1 and S2 were relatively close to each other, with a pairwise Bray-Curtis dissimilarity of 0.234, H1 was even closer to both severe-diarrhea samples, with dissimilarities of 0.223 to S1 and 0.177 to S2. By comparison, the H1-H2 and M1-M2 dissimilarities were 0.747 and 0.673, respectively. These results indicate substantial inter-individual variation and show that proximity in ordination space was not specific to the severe-diarrhea category.

### 3.3. Taxonomic Composition of the Fecal Bacterial Communities

Taxonomic profiling revealed substantial inter-individual variation in the fecal bacterial communities of the healthy and mildly diarrheic calves, whereas the two severe-diarrhea samples shared several taxonomic features despite the lack of clear severity-based separation in the PCoA (Figure 2). At the phylum level, *Proteobacteria* dominated both severe-diarrhea microbiomes, accounting for 86.76% and 84.25% of the bacterial-classified reads in S1 and S2, respectively. In contrast, Firmicutes represented only 1.86% and 3.29%, while Bacteroidetes accounted for 7.18% and 10.52%, respectively. Among the non-severe samples, *Proteobacteria* abundance varied markedly, ranging from 5.92% to 61.58% in the healthy calves and from 7.84% to 66.44% in the mildly diarrheic calves. Likewise, substantial variation was observed for Firmicutes and Bacteroidetes across the healthy and mild-diarrhea samples. Thus, the most consistent phylum-level feature observed in the present dataset was a *Proteobacteria*-dominated community accompanied by marked depletion of Firmicutes in both severe-diarrhea samples.

At the genus level, the fecal bacterial communities were also highly heterogeneous in the healthy and mild-diarrhea groups (Figure 2B). H1 was dominated by *Escherichia* (52.66%), whereas H2 was dominated by *Bacteroides*. In the mildly diarrheic group, M1 showed a predominance of *Lactobacillus*, whereas M2 contained relatively high proportions of *Escherichia* (28.56%) and *Pseudomonas*. By contrast, both severe-diarrhea samples were dominated by *Escherichia*, which accounted for 77.29% in S1 and 61.28% in S2. The proportion of *Shigella*-assigned reads was 5.22% in S1 and 6.09% in S2, while *Salmonella* was particularly enriched in S2 (7.07%). Overall, these data indicate that severe diarrhea was associated with a comparatively consistent taxonomic profile characterized by the dominance of *Proteobacteria* and a high abundance of *Escherichia*-related taxa, whereas the healthy and mildly diarrheic calves showed marked inter-individual heterogeneity.

### 3.4. Expansion of Enterobacteriaceae-Related Taxa and Depletion of Selected Anaerobic Taxa in Severe Diarrhea

**Figure 3:**
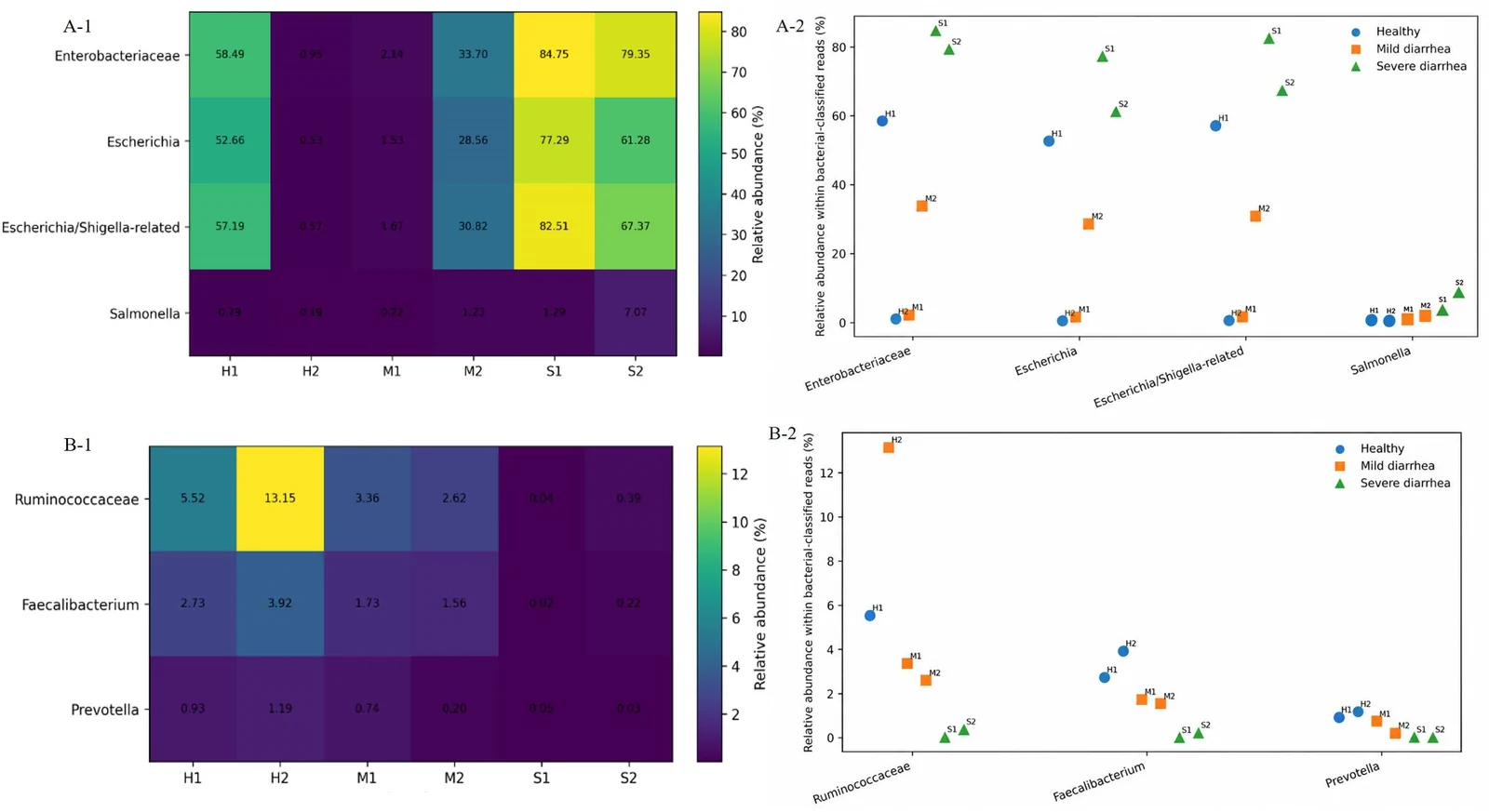
Expansion of *Enterobacteriaceae*-related taxa and depletion of selected anaerobic taxa in severe diarrhea. (**A-1**,**A-2**) Relative abundance of taxa enriched in the severe-diarrhea microbiomes. (**B-1**,**B-2**) Relative abundance of taxa depleted in the severe-diarrhea microbiomes.

Taxonomic resolution indicated that the *Proteobacteria*-dominated profiles of the severe-diarrhea samples were largely attributable to *Enterobacteriaceae*-related taxa (Figure 3(A-1,A-2)). *Enterobacteriaceae* accounted for 84.75% and 79.35% of the bacterial-classified reads in S1 and S2, respectively, compared with 0.95–58.49% among the four non-severe samples. Within this pattern, *Escherichia* represented 77.29% and 61.28% of the bacterial communities in S1 and S2, while combined *Escherichia*-/*Shigella*-related assignments reached 82.51% and 67.37%, respectively. *Salmonella*-assigned reads were also higher in both severe-diarrhea samples, particularly in S2 (7.07%). Moreover, H1 exhibited comparatively high *Enterobacteriaceae* and *Escherichia*-related abundance, highlighting the marked inter-individual heterogeneity present outside the severe-diarrhea group.

Several taxa prevalent in the non-severe microbiomes were markedly depleted in both severe-diarrhea samples (Figure 3(B-1,B-2)). *Ruminococcaceae* accounted for only 0.04% and 0.39% of bacterial-classified reads in S1 and S2, compared with 2.62–13.15% among the four non-severe samples. Likewise, *Faecalibacterium* represented only 0.02% and 0.22% in S1 and S2, compared with 1.56–3.92% in the non-severe samples. *Prevotella* also showed low abundance in the severe-diarrhea microbiomes (0.05% and 0.03%), whereas it ranged from 0.2% to 1.19% in the non-severe samples. These findings indicate that the severe-diarrhea microbiomes were characterized not only by expansion of *Enterobacteriaceae*-related taxa but also by depletion of several anaerobic gut bacterial groups.

**Figure A1:**
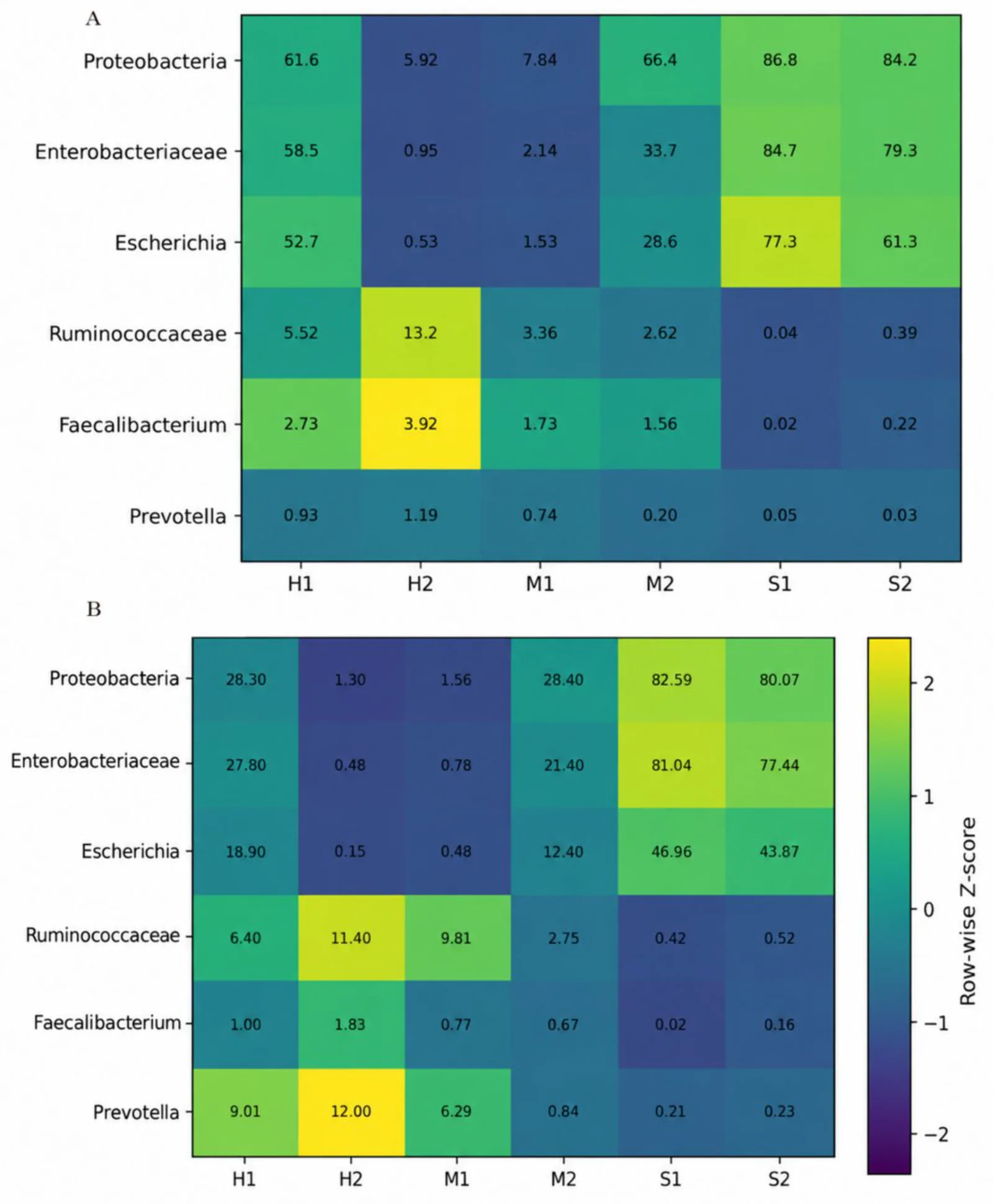
Concordance between read-based Kraken2 and gene-abundance-weighted NR taxonomic profiles. (**A**) Kraken2 read-based relative abundance of selected bacterial taxa. Percentages were calculated relative to the total number of reads classified as Bacteria within each sample. (**B**) NR gene-abundance-weighted relative abundance of the same selected taxa. For all NR taxonomic ranks, percentages were recalculated using the total normalized gene abundance assigned to Bacteria within each sample as a common denominator, including genes unresolved at lower taxonomic ranks. Cell labels show the unscaled relative abundance (%), whereas colors in panel B represent row-wise z-scores across the six samples.

To determine whether the major read-based taxonomic pattern was reproduced using an independent gene-centered approach, predicted genes were additionally assigned taxonomically against the NR database and weighted according to their estimated gene abundances. For all NR taxonomic ranks, the total abundance assigned to Bacteria within each sample was used as a common denominator, including genes unresolved at lower taxonomic ranks. After recalculation using this consistent denominator, Proteobacteria represented 82.59% and 80.07% of the gene-based bacterial profiles in S1 and S2, respectively, whereas *Enterobacteriaceae* represented 81.04% and 77.44%. Escherichia accounted for 46.96% and 43.87%, respectively. *Ruminococcaceae* (0.42% and 0.52%), *Faecalibacterium* (0.02% and 0.16%), and *Prevotella* (0.21% and 0.23%) remained at low abundance in S1 and S2 (Figure A1). Although relative-abundance estimates differed between the read-based Kraken2 and gene-abundance-weighted NR approaches, both methods supported the same overall taxonomic pattern in S1 and S2, characterized by high representation of *Enterobacteriaceae*-related taxa and low representation of selected anaerobic taxa.

### 3.5. Functional Profiles of the Severe-Diarrhea Microbiome Revealed by eggNOG/COG and KEGG Annotation

**Figure 4:**
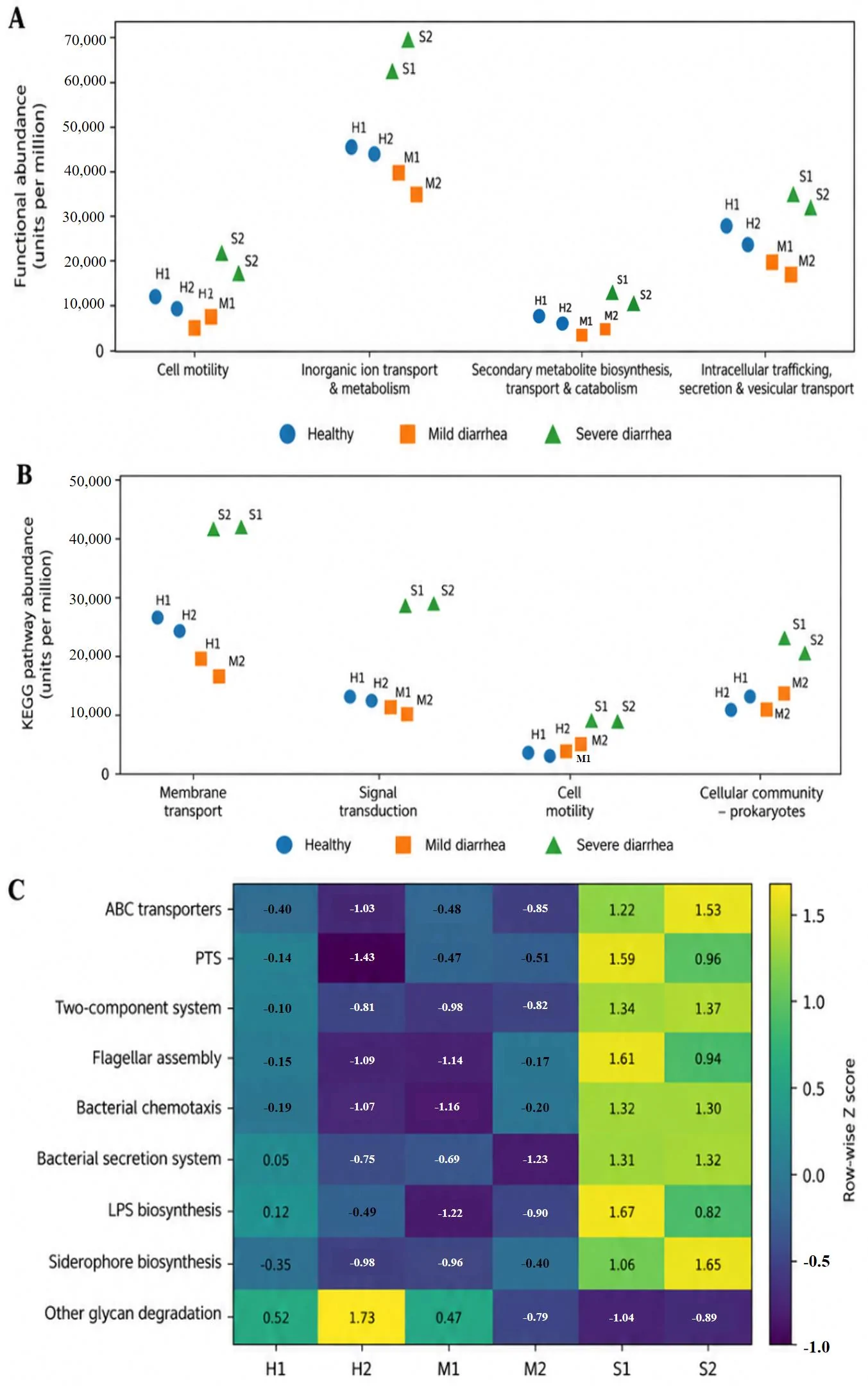
Selected eggNOG/COG and KEGG gene-content profiles across the six fecal metagenomes. (**A**) Selected eggNOG/COG functional categories, and (**B**) selected KEGG Level 2 categories. Healthy, mildly diarrheic, and severely diarrheic samples are shown as blue circles, orange squares, and green triangles, respectively. Abundances in panels A and B are expressed as normalized gene-abundance units per million. Displayed features were selected post hoc based on concordant directional differences between both severe-diarrhea samples and all four non-severe samples. (**C**) Row-wise Z-score heatmap of selected KEGG Level 3 pathways across the six samples (ddof = 0; no log transformation). Numerical annotations represent Z-scores.

eggNOG/COG annotation results (Figure 4A) showed that genes assigned to cell motility (N), inorganic ion transport and metabolism (P), secondary metabolite biosynthesis, transport and catabolism (Q), and intracellular trafficking, secretion and vesicular transport (U) were consistently more abundant in S1 and S2 than in each of the four non-severe samples. These categories were selected for focused presentation because they showed a concordant directional pattern in both severe-diarrhea samples relative to all four non-severe samples, according to the descriptive screening criterion described in the Methods. The complete six-sample COG abundance matrix is provided in Supplementary Tables S1–S7. Among these categories, cell motility showed the strongest relative increase, with the mean abundance in the two severe-diarrhea samples approximately 2.5-fold that of the four non-severe samples.

In KEGG Level 2, both severe-diarrhea samples exhibited higher abundances of genes assigned to membrane transport, signal transduction, cell motility, and cellular community-prokaryotes than all four healthy or mildly diarrheic samples (Figure 4B). Membrane transport abundance reached 40,916.03 and 41,804.50 normalized gene-abundance units per million in S1 and S2, respectively, whereas signal transduction reached 27,498.57 and 27,803.69 units per million. Cell motility and cellular community-prokaryotes displayed similar patterns, with both severe-diarrhea samples exceeding all four non-severe samples. In KEGG Level 3, a concordant gene-content pattern was observed in S1 and S2 (Figure 4C). Eight selected pathways showed higher abundance in both severe-diarrhea samples than in each of the four non-severe samples. These included ABC transporters, the phosphotransferase system (PTS), two-component systems, flagellar assembly, bacterial chemotaxis, bacterial secretion systems, lipopolysaccharide biosynthesis, and biosynthesis of siderophore-group nonribosomal peptides. In contrast, other glycan degradation showed the opposite pattern, with abundance values of 1487.94 and 1748.78 in S1 and S2, respectively, lower than those observed in all four non-severe samples. These results indicate that the severe-diarrhea fecal microbiomes were characterized by a shift in microbial function toward environmental sensing, substrate transport, motility, bacterial community-associated processes, and cell-envelope-related functions, accompanied by reduced representation of genes assigned to glycan degradation. These patterns represent differences in metagenomic gene-content potential and should not be interpreted as evidence of pathway activation, gene expression, or metabolic activity.

### 3.6. CAZyme Family Profiles Revealed Carbohydrate- and Cell-Envelope-Associated Functional Potential

**Figure 5:**
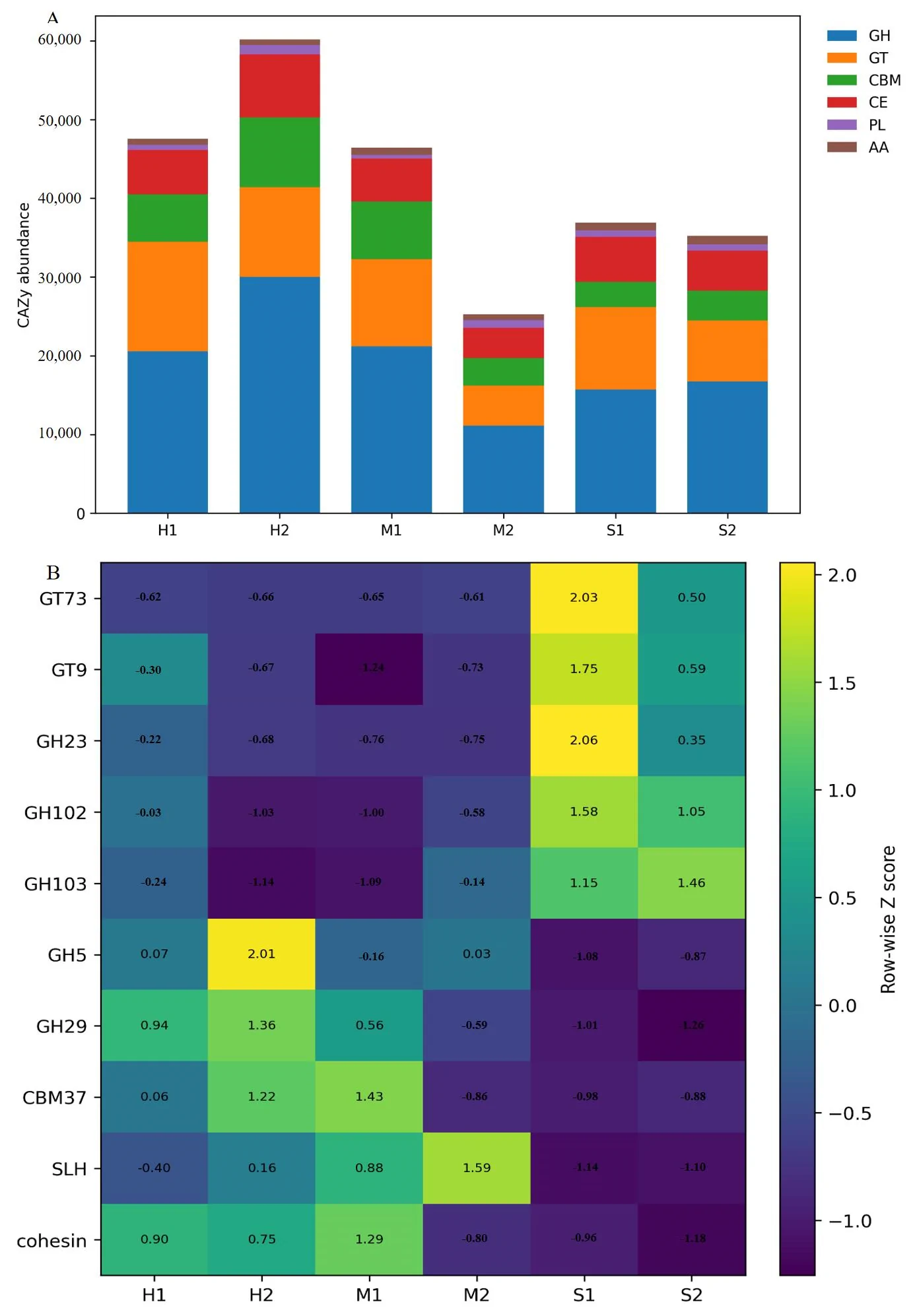
CAZy gene-content profiles of fecal microbiomes from healthy, mildly diarrheic, and severely diarrheic calves. (**A**) Abundance profiles of the recovered broad CAZy classes. (**B**) Selected CAZy families showing concordant directional differences between both severe-diarrhea samples and all four non-severe samples. Abundance values are expressed as normalized gene-abundance units per million. Displayed family-level features were selected post hoc for descriptive visualization and were not defined by statistical significance testing. Heatmap colors represent row-wise Z-scores across the six individual samples (ddof = 0; no log transformation), whereas cell labels show Z-score values.

CAZy annotation showed that glycoside hydrolases (GHs), glycosyltransferases (GTs), carbohydrate-binding modules (CBMs), and carbohydrate esterases (CEs) represented the major CAZyme classes across the six fecal metagenomes, whereas polysaccharide lyases (PLs) and auxiliary activities (AAs) were less abundant (Figure 5A). Among these classes, GH was the most abundant CAZy class, with abundance values ranging from 11,123.14 to 30,037.50. At the family level, a more consistent pattern emerged in the severe-diarrhea microbiomes (Figure 5B). The displayed CAZy families were selected for focused presentation because they showed concordant directional differences in both severe-diarrhea samples relative to all four non-severe samples, according to the same descriptive screening criterion described in the Methods. Several CAZy families associated with bacterial cell-envelope biosynthesis or remodeling showed higher abundance in both severe-diarrhea samples. In particular, GT73 showed markedly high abundance in S1 and S2 (157.86 and 67.96, respectively), whereas the four non-severe samples showed only 0.17–3.06. GT9 was also enriched in the severe-diarrhea samples (897.54 and 567.36 in S1 and S2), as were GH23 (2685.34 and 1431.19), GH102 (156.79 and 125.92), and GH103 (132.32 and 150.12). These results indicate increased representation of several CAZyme families linked to lipopolysaccharide-associated glycosyltransferase activity and peptidoglycan remodeling in the severe-diarrhea microbiomes. Conversely, several CAZy families associated with complex carbohydrate utilization showed lower abundance in the severe-diarrhea samples. These families include GH5, which was 38.99 and 74.41 in S1 and S2, respectively, and 196.93–573.55 in the non-severe diarrhea samples; GH29, which was 231.10 and 139.25 in S1 and S2, respectively, and 393.64–1139.32 in the non-severe diarrhea samples; and CBM37, which was 8.16 and 16.03 in S1 and S2, respectively, and 17.19–194.53 in the non-severe diarrhea samples. Cellulosome-associated features were markedly reduced, as indicated by the low abundance of SLH and cohesin in the severe-diarrhea samples. These findings suggest that severe diarrhea was associated with remodeling of the carbohydrate-active gene-content repertoire, characterized by reduced representation of several complex-polysaccharide-associated functions and increased representation of bacterial cell-envelope-associated functions. Because these results are based on metagenomic gene abundance, they represent encoded functional potential and do not demonstrate enzyme activity or metabolic flux in vivo.

### 3.7. CARD-Associated Sequence-Homology Candidates Across the Six Fecal Metagenomes

Re-evaluation of the archived CARD homology output identified 163 unique catalog query genes meeting the revised screening criteria of ≥99% amino-acid identity and ≥95% alignment-length ratio. Thirty query genes whose CARD reference labels explicitly indicated mutation-dependent resistance were excluded because the required resistance-associated mutations could not be verified. The final screened set therefore contained 133 distinct catalog query genes corresponding to 120 ARO identifiers. These entries were interpreted as CARD-associated sequence-homology candidates rather than distinct resistance types, CARD/RGI-validated resistant alleles, or phenotypically confirmed antimicrobial-resistance determinants.

**Figure A2:**
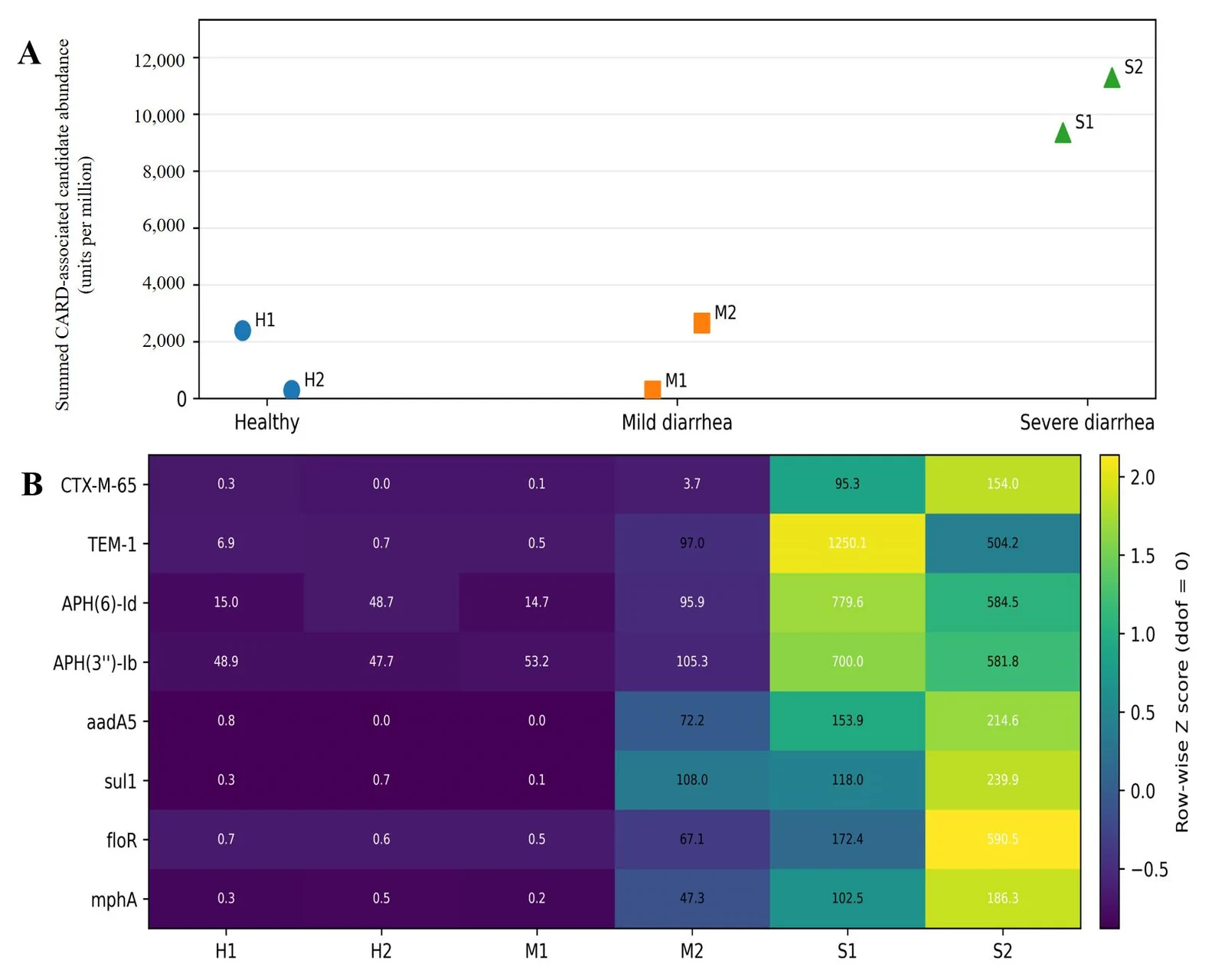
CARD-associated candidate profiles across the six fecal metagenomes. (**A**) Summed normalized abundance of the 138 retained CARD-associated candidates after conservative rescreening. (**B**) Row-wise Z-score heatmap of eight selected CARD reference labels that showed higher abundance in both severe-diarrhea samples than in each of the four non-severe samples. Candidate abundances are expressed as normalized gene-abundance units per million. Heatmap colors represent row-wise Z-scores across the six individual samples (ddof = 0; no log transformation), whereas numerical annotations show the unscaled abundance values. Feature selection was performed post hoc for descriptive visualization and was not based on statistical significance testing. The retained candidates represent sequence-homology-based antimicrobial-resistance potential rather than CARD/RGI-validated resistant alleles or phenotypically confirmed antimicrobial resistance.

Using the six-sample normalized gene-abundance matrix, the summed abundance of the 133 retained candidates was 2169.16, 285.35, 251.32, 2591.17, 8777.27, and 10,815.40 normalized gene-abundance units per million in H1, H2, M1, M2, S1, and S2, respectively (Figure A2A). Thus, the two severe-diarrhea samples showed the highest summed abundance of retained CARD-associated candidates among the six samples. The numbers of retained candidates with non-zero abundance were 59, 76, 66, 120, 65, and 98 in H1, H2, M1, M2, S1, and S2, respectively. Therefore, the greater summed abundance in S1 and S2 was not accompanied by a corresponding increase in the number of detected candidates; notably, M2 contained the largest number of non-zero candidates despite a substantially lower summed abundance than either severe-diarrhea sample.

For focused descriptive visualization, eight CARD reference labels—*CTX-M-65*, *TEM-1*, *APH(6)-Id*, *APH(3″)-Ib*, *aadA5*, *sul1*, *floR*, and *mphA*—were selected because each showed higher abundance in both S1 and S2 than in each of the four non-severe samples (Figure A2B). These patterns represent sequence-homology-based antimicrobial-resistance potential and should not be interpreted as evidence of gene expression, phenotypically confirmed antimicrobial resistance, or resistance attributable to a particular bacterial host.

## 4. Discussion

The study indicates that severe diarrhea in calves was accompanied by coordinated changes in fecal microbial community structure and metagenomic functional potential. The most informative feature was not a single bacterial taxon or pathway but the coexistence of reduced bacterial diversity, pronounced *Enterobacteriaceae*-related dominance, depletion of several anaerobic bacterial groups, shifts in transport, motility, secretion, and cell-envelope-associated functions, remodeling of carbohydrate-active enzyme families, and increased representation of selected antimicrobial-resistance-associated genes. The two severe-diarrhea samples showed relatively concordant profiles across these complementary taxonomic and functional annotation layers, although the ordination analysis did not support a distinct community cluster defined by clinical severity. Reduced microbial diversity during calf diarrhea has been reported repeatedly, although its magnitude and taxonomic context vary among studies. Oikonomou et al. linked early-life fecal microbial composition, particularly *Faecalibacterium*, with calf health and growth [5]. Gomez et al. subsequently demonstrated differences in community structure between healthy and diarrheic calves but also showed that both the healthy microbiota and diarrhea-associated alterations were farm dependent [6]. Studies by Kim et al., Hennessy et al., and Gomez et al. further demonstrated that diarrhea is frequently accompanied by lower diversity and marked changes in microbial composition, while the specific taxa involved differ across cohorts and management systems [7,8,9]. Longitudinal observations are particularly relevant because they show that microbial diversity and community structure can change rapidly around the onset of diarrhea and partly recover after clinical resolution [10]. These findings provide an important context for the present PCoA results. The PCoA should not be interpreted as evidence of a community structure specific to severe diarrhea. Although S1 and S2 were relatively close in ordination space, H1 was even closer to both severe-diarrhea samples based on Bray–Curtis dissimilarity. Thus, the ordination did not show clear separation according to clinical severity and instead highlighted substantial inter-individual variation within this small cohort. The lower bacterial diversity and *Enterobacteriaceae*-related dominance observed in both severe-diarrhea samples were broadly consistent with previous reports of calf diarrhea-associated dysbiosis and were therefore biologically plausible. In contrast, the close ordination of the clinically healthy H1 sample with S1 and S2 was unexpected and argues against a simple severity-dependent community pattern. The taxonomic similarities shared by S1 and S2 should therefore be interpreted as descriptive patterns rather than as evidence of a discrete severe-diarrhea microbiome state. Age, diet, environment, management, pathogen exposure, and individual microbial succession can all influence the developing calf microbiota [6,7,8,9,10]. The most prominent taxonomic characteristic shared by the severe-diarrhea samples was expansion of *Enterobacteriaceae*-related taxa together with loss of selected anaerobic populations. This pattern agrees closely with the large meta-omics study of Whon et al., in which calf diarrhea was associated with prolonged expansion of autochthonous *Enterobacteriaceae* and accompanying functional dysbiosis [11]. A recent multi-farm metagenomic investigation in Inner Mongolia also identified pronounced community disruption across diarrhea severities and implicated particular *Escherichia coli* (*E. coli*) genomic populations in severe disease [13]. The reduction of *Ruminococcaceae*, *Faecalibacterium*, and *Prevotella* in the present severe samples is likewise consistent with studies associating these or related anaerobic taxa with healthier calf intestinal communities [5,6,9]. The agreement between the read-based Kraken2 profiles and the independent gene-abundance-weighted NR assignments strengthens confidence in the direction of this community shift, although the relative-abundance estimates differed between the two approaches because short-read classification and gene-based taxonomic assignment quantify microbial communities using different analytical units.

The results of this study suggest a biologically plausible ecological framework between the decline of obligate anaerobes and the expansion of facultative anaerobes. Experimental studies in other mammalian systems have shown that inflammation-derived nitrate can provide a respiratory advantage to *E. coli* [16], whereas depletion of butyrate-producing Clostridia can increase epithelial oxygenation and favor the expansion of facultative *Enterobacteriaceae* [17]. These mechanisms were not directly measured in the present study and are therefore considered biologically plausible explanations rather than demonstrated mechanisms. The reverse direction is also possible; intestinal infection or other disease processes may first disrupt the gut environment, with microbiota restructuring emerging as a consequence. This uncertainty remains a central issue in calf diarrhea research. The COG and KEGG profiles provided complementary gene-content perspectives, with higher representation of functions related to environmental sensing, transport, motility, secretion, and Gram-negative cell-envelope processes in the severe-diarrhea samples. These annotations are biologically coherent with the expansion of Gram-negative facultative bacteria. Flagella and chemotaxis allow bacteria to respond to spatial changes in nutrients and environmental conditions and can contribute to colonization behavior in E. coli [18]. Two-component systems provide a major mechanism through which bacteria sense and respond to environmental stimuli, while secretion systems mediate diverse interactions between Gram-negative bacteria and their surroundings or host cells [19,20]. Increased representation of genes involved in LPS biosynthesis is also compatible with a community dominated by Gram-negative taxa because LPS is a major component of the Gram-negative outer membrane [21]. Siderophore-associated functions fit the same ecological framework, as iron acquisition is an important competitive trait under iron-limited conditions [22]. The co-occurrence of *Enterobacteriaceae* expansion and these gene-content patterns is consistent with a community-composition-driven change in functional potential. The higher representation of GT73 and GT9 in the severe samples is noteworthy because characterized members of these families include glycosyltransferases involved in Kdo transfer and LPS-core assembly; GH23, GH102, and GH103 include enzymes involved in peptidoglycan hydrolysis or lytic transglycosylase activity, linking their increased representation to bacterial cell-wall turnover and remodeling [23,24]. This CAZy pattern provides a complementary annotation perspective that is directionally consistent with the KEGG gene-content profile for Gram-negative cell-envelope-associated potential. Importantly, the COG, KEGG, and CAZy analyses were all derived from the same metagenomic gene content and therefore provide complementary, rather than independent, functional annotations. Higher abundance of genes assigned to a pathway or enzyme family indicates greater representation of the corresponding encoded potential but does not demonstrate transcriptional activation, enzyme activity, metabolite production, or increased metabolic flux. At the other end of the functional spectrum, lower GH5, GH29, CBM37, SLH, and cohesin abundance suggests loss of selected carbohydrate-processing capacities associated with anaerobic populations. CBM37 has been characterized in Ruminococcus albus as part of a specialized system for carbohydrate binding and cell-surface attachment of glycoside hydrolases [25]. The parallel depletion of *Ruminococcaceae* and several carbohydrate-associated CAZy families therefore supports a broader ecological shift away from some anaerobic carbohydrate-utilizing populations. The CARD analysis provided an additional view of antimicrobial-resistance potential in the six fecal metagenomes. After conservative filtering of the historical homology-based annotations, several CARD-associated candidates were more abundant in S1 and S2, including genes associated with resistance to β-lactams, aminoglycosides, sulfonamides, phenicols, and macrolides. Previous studies have shown that the calf resistome changes substantially during early life and is associated with microbiota development, diet, and antimicrobial exposure [14,26,27]. In the present study, H1 and H2 had not received treatment before sampling, whereas M1, M2, S1, and S2 had been treated for diarrhea. Nevertheless, the highest summed abundance of retained CARD-associated candidates occurred in S1 and S2 rather than in the two mild-diarrhea samples. Thus, the observed resistome pattern did not simply correspond to the documented treatment status. The marked abundance of *Enterobacteriaceae*-related taxa in S1 and S2 may have contributed to the higher representation of resistance-associated genes, although the present short-read data do not establish the bacterial hosts of individual resistance determinants. Because the specific drugs administered to each diarrheic calf and the interval between treatment and sampling were unavailable, the relative contributions of treatment, clinical status, and microbial community composition cannot be separated in this dataset.

Overall, the taxonomic and functional analyses indicated coordinated differences in community composition and encoded functional potential in the two severe-diarrhea samples. However, several limitations should be considered. First, the study included only six calves, with two animals in each clinical category; therefore, the observed differences should be regarded as descriptive and exploratory rather than as population-level or severity-specific microbiome signatures. Second, unequal read-retention rates after quality filtering may have affected the detection of low-abundance features, and the archived quality-control outputs did not allow the lower retention in individual samples to be attributed to a specific filtering step. Third, the four diarrheic calves had received treatment before sampling, but detailed individual treatment regimens were unavailable; therefore, treatment effects could not be separated from differences associated with clinical status. In addition, the etiology of diarrhea could not be established from genus-level metagenomic assignments alone, and Escherichia/Shigella assignments should be interpreted cautiously because short-read classification cannot reliably distinguish these closely related taxa. Finally, the CARD results represent resistance-associated genetic potential inferred from sequence homology rather than phenotypically confirmed resistance. Future studies should therefore include larger longitudinal cohorts with standardized clinical grading, pathogen testing, detailed treatment records, and preferably pretreatment or untreated comparisons. Phenotypic antimicrobial-susceptibility testing would also be valuable for validating sequence-based resistance predictions.

## 5. Study Contributions and Implications

The principal contribution of this study is the integrated characterization of multiple metagenomic layers within the same small cohort. Read-based and gene-abundance-weighted taxonomic profiles were considered together with COG/KEGG and CAZy gene-content profiles and conservatively screened CARD-associated resistance potential. Relative to previous multi-farm work in Inner Mongolia [13], the present study contributes a sample-resolved integration of read-based and gene-abundance-weighted taxonomic profiles with COG/KEGG, CAZy, and CARD-associated gene-content patterns within the same metagenomic dataset. The concordant cross-layer patterns observed in the two severe-diarrhea samples provide hypothesis-generating evidence for relationships among community composition, encoded functional potential, and antimicrobial-resistance-associated genetic potential that can be tested in larger longitudinal cohorts.

## Data Availability

The original contributions presented in this study are included in the article/Supplementary Materials. Further inquiries can be directed to the corresponding authors.

## Supplementary Materials

The following supporting information can be downloaded at: https://www.mdpi.com/article/10.3390/microorganisms14102370/s1, Table S1. Complete eggNOG/COG functional-category abundance matrix-COG; Table S2. Complete KEGG Level 1 abundance matrix; Table S3. Complete KEGG Level 2 abundance matrix; Table S4. Complete KEGG Level 3 abundance matrix; Table S5. Complete CAZy class-level abundance matrix.; Table S6. Complete CAZy family-level abundance matrix; Table S7. Figure 4 and Figure 5 consistency audit.

## Appendix A

**Table A1:** Available clinical and management information for the six calves.

Sample Name	Clinical Group	Age	Weight	Breed	Location	Fecal Status	Management	Treatment Before Sampling
H1	Healthy	2 months old	30 ± 2 kg	Simmental	Chahar Right Middle Banner, Ulanqab, Inner Mongolia	Normal	Grazing	No
H2	Healthy							
M1	Mild					loose or mucus-containing feces		β-lactams, cephalosporins, aminoglycosides, macrolides, tetracyclines, fluoroquinolones, amphenicols, and lincosamides, together with supportive therapies
M2	Mild							
S1	Severe					watery feces		
S2	Severe							

**Table A2:** Assembly statistics for the six fecal metagenomic samples.

Sample Name	Contigs	Total Length (bp)	N50 (bp)	N90 (bp)	Max (bp)	Min (bp)
H1	199,424	389,347,804	3703	689	351,405	500
H2	81,029	155,032,825	4104	662	309,569	500
M1	131,905	228,126,171	2924	642	255,724	500
M2	23,783	31,164,891	1667	579	63,213	500
S1	33,185	72,989,736	6698	689	620,261	500
S2	26,869	42,099,836	2547	607	281,855	500

**Table A3:** ORF prediction statistics for the six fecal metagenomic samples.

Sample Name	ORFs	Total ORF Length (bp)	Mean Length (bp)	Max (bp)	Min (bp)
H1	513,547	348,308,796	678.24	23,825	60
H2	192,888	138,995,286	720.6	15,844	60
M1	301,113	206,586,472	686.08	20,140	60
M2	43,458	26,344,749	606.21	7269	60
S1	91,381	65,573,784	717.59	21,074	60
S2	53,162	36,615,664	688.76	9892	60

**Table A4:** Software, versions, parameters, and database information used in the bioinformatic analyses.

Analysis	Software	Version	Parameters
QC	Trimmomatic	0.36	ILLUMINACLIP …
Taxonomy	Kraken2	2.0.6	--threads 16 …
Assembly	MEGAHIT	1.1.1-2-g02102e1	--min-contig-len 500
Gene catalog	CD-HIT	4.8.1	-n 9 -c 0.95 …
Gene abundance	Salmon	1.1.0	default
NR	DIAMOND	0.9.22.123	blastp --evalue 0.00001
eggNOG	eggNOG-mapper	v5.0	default
KEGG	KofamScan	1.2.0	default
CARD	BLASTP	2.9.0+	-max_target_seqs 5 -evalue 1 × 10^−50^ …
CAZy	hmmscan	dbCAN-HMMdb-V8	default

**Table A5:** Sequencing quality-control, host-filtering, and taxonomic-classification statistics for the fecal samples.

Sample Name	Raw Reads	Clean Reads	Raw-to-Clean Retention (%)	Clean Q20 (%)	Clean Q30 (%)	Clean GC (%)	Host-Derived (%)	Non-Host (%)	Bacterial (%)	Unclassified (%)
H1	126,397,152	108,566,340	85.89	96.60	88.30	44.36	15.75	84.25	45.53	54.05
H2	96,815,376	67,641,094	69.87	97.58	92.09	47.51	54.02	45.98	41.77	57.96
M1	85,847,754	65,320,692	76.09	97.14	90.80	43.66	53.78	46.22	36.87	62.74
M2	66,296,600	19,745,986	29.78	97.80	92.86	48.07	73.48	26.52	34.56	64.79
S1	132,347,932	114,949,426	86.85	96.92	89.39	46.16	30.54	69.46	80.03	18.65
S2	67,233,622	28,099,062	41.79	97.91	93.13	49.27	57.97	42.03	75.09	23.43

Note: Raw-to-clean retention was calculated from individual read counts. Host-derived and non-host proportions were calculated from paired-read counts at the host-filtering stage. Bacterial and unclassified proportions were calculated relative to the total read pairs entering downstream taxonomic classification. Percentages from different processing stages therefore use different denominators.
