# Supplementary material for: Exploratory Shotgun Metagenomic Analysis of the Fecal Microbiome and Antimicrobial-Resistance Potential in Simmental Calves with Different Diarrheal Statuses
Source: Microorganisms. 2026 Oct 16;14(10):2370. doi: 10.3390/microorganisms14102370 (PMC13707887; doi:10.3390/microorganisms14102370)
Supplement: Supplementary file 1 [file microorganisms-14-02370-s001.zip › microorganisms-4600848-supplementary.pdf]

Supplementary Table S1. Complete eggNOG/COG functional-category abundance matrix-COG

Feature	Description / activity	H1	H2	M1	M2	S1	S2
A	RNA processing and modification	233.207978	222.273312	140.48845	164.800881	264.047357	312.909697
B	Chromatin structure and dynamics	54.128179	21.358735	47.064454	88.424707	241.115614	22.141644
C	Energy production and conversion	44185.89174	45789.62787	42384.18541	40600.43137	49968.06096	56168.79896
D	Cell cycle control, cell division, chromosome partitioning	14776.56659	12516.18949	15874.71037	10486.6292	10358.26619	10588.52025
E	Amino acid transport and metabolism	50056.05366	53408.79501	56284.63522	45382.1868	54429.07856	63137.09439
F	Nucleotide transport and metabolism	32696.46099	26953.67978	34078.74496	23782.90116	38186.05408	42035.65662
G	Carbohydrate transport and metabolism	56472.29141	64629.40234	59726.26926	41248.41124	57507.75832	62538.61546
H	Coenzyme transport and metabolism	31293.50794	38777.94023	34115.23198	24280.76898	27960.76955	32782.95708
I	Lipid transport and metabolism	18699.61536	17822.47769	17218.90698	15288.50121	18075.86323	21395.64257
J	Translation, ribosomal structure and biogenesis	48950.20691	43018.63897	53980.28611	35657.25476	40172.64468	41762.60338
K	Transcription	63389.54385	53067.37036	56290.69361	44219.00539	65603.30382	55523.63041
L	Replication, recombination and repair	89781.75841	107000.8503	120337.9029	175777.1269	66708.76671	108366.7742
M	Cell wall/membrane/envelope biogenesis	68534.12623	69832.23265	62709.79915	42708.48898	60269.08328	54692.69696

N	Cell motility	13205.32553	4795.136807	4967.904984	8772.397813	23362.69633	16278.19469
O	Posttranslational modification, protein turnover, chaperones	24106.177	20564.03739	21842.6425	16759.35813	22095.41306	23402.00542
P	Inorganic ion transport and metabolism	47673.7519	46477.91035	43334.14828	36467.91917	63211.17564	68662.80559
Q	Secondary metabolites biosynthesis, transport and catabolism	8340.719967	7107.410198	6407.071197	8678.803215	11286.87268	12835.98715
R	General function prediction only	0	0	0	0	0	0
S	Function unknown	129510.0254	117966.971	122051.2192	87243.94467	105988.952	96267.93945
T	Signal transduction mechanisms	28331.18539	29924.1601	25039.34653	22353.96077	25635.11161	25416.79991
U	Intracellular trafficking, secretion, and vesicular transport	29712.92493	22468.06045	20777.18467	19534.0504	36554.12956	32924.28703
V	Defense mechanisms	20010.52608	21475.6702	23455.96734	13162.71099	13523.54894	13749.30703
W	Extracellular structures	119.895605	39.548326	274.796177	158.225407	354.468323	219.789129
Y	Nuclear structure	0	0.287463	0	0	0	0.795125
Z	Cytoskeleton	207.030513	143.18255	192.483253	126.234851	120.620998	134.569839

Supplementary Table S2. Complete KEGG Level 1 abundance matrix

**Feature**	**Description / activity**	**H1**	**H2**	**M1**	**M2**	**S1**	**S2**
1. Metabolism	Metabolism	143582.7441	142535.0817	141470.3438	111378.4857	169253.4944	185811.7526
2. Genetic Information Processing	Genetic Information Processing	46758.39682	43905.18181	55266.84254	33917.33557	36026.73383	38327.70368
3. Environmental Information Processing	Environmental Information Processing	41799.88634	28745.26691	34144.7728	30732.53475	67412.19172	68478.49943
4. Cellular Processes	Cellular Processes	26321.00719	20566.36024	22075.426	18786.15271	35219.38833	32649.0441

5. Organismal Systems	Organismal Systems	7164.667126	6825.002552	7449.246164	5272.07046	7065.43636	7823.006529
6. Human Diseases	Human Diseases	20952.45572	16464.79795	18782.61783	13297.21899	26173.8433	22342.05136

Supplementary Table S3. Complete KEGG Level 2 abundance matrix

Feature	Description / activity	H1	H2	M1	M2	S1	S2
1.0 Global and overview maps	Global and overview maps	132978.7022	134072.8407	132337.8267	104236.6908	155978.1384	171720.4236
1.1 Carbohydrate metabolism	Carbohydrate metabolism	45730.90444	45447.30424	43970.4585	33782.66038	56996.75492	61214.88573
1.10 Biosynthesis of other secondary metabolites	Biosynthesis of other secondary metabolites	8273.621293	9835.208121	7636.49794	6221.285802	7806.800447	8619.746816
1.11 Xenobiotics biodegradation and metabolism	Xenobiotics biodegradation and metabolism	5519.73314	4432.637936	5352.062632	4122.711827	7259.699919	8632.332398
1.2 Energy metabolism	Energy metabolism	21982.45867	22904.06157	22833.27393	21288.14849	25104.07818	29195.07602
1.3 Lipid metabolism	Lipid metabolism	10732.85029	10842.06192	11072.92794	7850.471421	12267.04804	14018.90602
1.4 Nucleotide metabolism	Nucleotide metabolism	18724.86842	17820.44079	21395.52009	13102.92926	19563.81302	20736.9454
1.5 Amino acid metabolism	Amino acid metabolism	32306.8029	35349.50673	33987.25546	27436.56096	35427.67322	41855.79322
1.6 Metabolism of other amino acids	Metabolism of other amino acids	10009.32039	9980.097469	9893.929445	8290.026802	11910.88654	15259.38064
1.7 Glycan biosynthesis and metabolism	Glycan biosynthesis and metabolism	16063.56804	16780.50784	14668.74213	8786.221607	14349.34016	13609.93956
1.8 Metabolism of cofactors and vitamins	Metabolism of cofactors and vitamins	26223.63671	27372.22249	25363.01113	21379.49259	32156.2623	34764.57754
1.9 Metabolism of terpenoids and polyketides	Metabolism of terpenoids and polyketides	5545.863284	5552.17854	5515.390117	3877.174328	6094.037388	7001.873583

2.1 Transcription	Transcription	1421.678951	1455.791483	1894.914476	1573.445974	920.141783	1147.408246
2.2 Translation	Translation	21005.35618	19378.12019	25672.44677	15499.48421	14342.63951	14697.03594
2.3 Folding, sorting and degradation	Folding, sorting and degradation	9840.015915	8432.761148	10479.22245	6544.613791	8995.613494	9798.207322
2.4 Replication and repair	Replication and repair	14491.58837	14638.50898	17220.25884	10299.7916	11768.33905	12685.05217
3.1 Membrane transport	Membrane transport	24293.3522	16166.97814	21969.86065	18425.36763	40916.027	41804.49674
3.2 Signal transduction	Signal transduction	18221.30621	13239.85977	12682.66847	12827.25573	27498.57287	27803.69467
3.3 Signaling molecules and interaction	Signaling molecules and interaction	66.446873	57.308068	55.964774	67.632362	4.15839	2.565909
4.1 Transport and catabolism	Transport and catabolism	3138.858489	3870.507395	2527.504955	1528.495806	2015.073551	2158.544123
4.2 Cell growth and death	Cell growth and death	6121.357466	6196.96822	6739.870863	4241.561965	4138.362298	4669.222909
4.4 Cellular community - prokaryotes	Cellular community - prokaryotes	15301.52953	10711.85132	13222.34494	10567.11689	22799.18494	20706.04852
4.5 Cell motility	Cell motility	3125.844395	683.695436	533.157353	3072.300954	7613.871363	6293.505304
5.1 Immune system	Immune system	922.100962	741.045047	721.770033	575.428664	685.489008	638.200335
5.10 Environmental adaptation	Environmental adaptation	1671.608919	1396.860798	1468.743462	1106.758858	1623.280348	1671.37189
5.2 Endocrine system	Endocrine system	2673.565321	2381.817768	3134.036478	2002.520011	2688.807754	2982.478833
5.3 Circulatory system	Circulatory system	0.786676	4.357608	2.408466	57.33117	13.190163	12.909639
5.4 Digestive system	Digestive system	369.445541	422.488743	195.918055	219.066413	369.460008	438.323857
5.5 Excretory system	Excretory system	200.847165	150.58128	168.743865	85.571875	282.601562	345.563398
5.6 Nervous system	Nervous system	799.164726	1092.665909	942.5965	776.042344	855.555368	1061.483053
5.8 Development and regeneration	Development and regeneration	110.486018	204.432852	75.897598	41.448816	28.380193	41.086111
5.9 Aging	Aging	1952.581113	1717.776381	2008.910499	1305.558552	1855.976021	1928.905601
6.1 Cancer: overview	Cancer: overview	2814.337566	2274.576788	3033.072786	1881.075055	2642.402932	2679.792188

6.10 Infectious disease: parasitic	Infectious disease: parasitic	229.895027	71.666512	244.395375	86.049713	325.832497	257.466603
6.11 Drug resistance: antimicrobial	Drug resistance: antimicrobial	8081.431637	6742.408607	7698.080027	5336.711723	10759.18508	10038.89493
6.12 Drug resistance: antineoplastic	Drug resistance: antineoplastic	1858.913312	1944.246681	2239.717584	1284.76935	2652.726002	1929.217014
6.2 Cancer: specific types	Cancer: specific types	685.424425	363.831508	410.859416	412.778997	947.485773	1009.651715
6.3 Immune disease	Immune disease	464.167653	536.340104	522.873618	254.511822	354.179786	308.727718
6.4 Neurodegenerative disease	Neurodegenerative disease	947.931449	861.482454	697.255202	727.12049	1061.455577	1053.294279
6.5 Substance dependence	Substance dependence	0.240188	1.481784	1.007415	9.74494	0.142988	3.893036
6.6 Cardiovascular disease	Cardiovascular disease	1276.122027	1057.603055	985.50032	871.72204	1202.295074	1302.719389
6.7 Endocrine and metabolic disease	Endocrine and metabolic disease	1766.320071	1645.962219	2073.721311	1251.901717	1627.480287	1809.085694
6.8 Infectious disease: bacterial	Infectious disease: bacterial	6461.913329	3779.343225	4514.352034	3555.504638	8326.296527	5622.911068
6.9 Infectious disease: viral	Infectious disease: viral	481.212735	447.071901	507.971732	398.281116	335.727297	316.557301

Supplementary Table S5. Complete CAZy class-level abundance matrix

Feature	H1	H2	M1	M2	S1	S2
AA	742.818235	691.179907	961.422314	715.771563	1010.13915	1089.645183
CBM	6013.747336	8830.642241	7350.22173	3507.324001	3171.068191	3779.379136
CE	5642.837526	8031.838557	5441.531625	3849.059274	5743.645107	5096.303397
GH	20554.6773	30037.49634	21178.56713	11123.14251	15724.54917	16731.25618
GT	13910.55506	11382.18073	11067.09863	5080.88328	10477.95155	7746.471949
PL	686.900621	1187.01523	455.04858	962.734216	771.25018	783.636736

SLH	636.876599	1089.313588	1667.447346	2243.118078	40.934003	71.393226
cohesin	103.10881	95.510815	121.926752	20.485943	12.86589	2.0496
dockerin	70.191615	80.041326	65.016544	13.339888	14.572676	11.833768
